# A survey of obstetric ultrasound uses and priorities for artificial intelligence-assisted obstetric ultrasound in low- and middle-income countries

**DOI:** 10.1038/s41598-025-87284-1

**Published:** 2025-01-31

**Authors:** Amy Sarah Ginsburg, Zylee Liddy, Eren Alkan, Kayla Matcheck, Susanne May

**Affiliations:** 1 Clinical Trials Center, University of Washington, Building 29, Suite 250, 6200 NE 74th Street, Seattle, WA 98115 USA; 2 Caption Health, GE HealthCare, San Mateo, CA USA

**Keywords:** Obstetric ultrasound, Low- and middle-income countries, Point of care, Artificial intelligence, Health care, Medical research

## Abstract

Obstetric ultrasound (OBUS) is recommended as part of antenatal care for pregnant individuals worldwide. To better understand current uses of OBUS in low- and middle-income countries and perceptions regarding potential use of artificial intelligence (AI)-assisted OBUS, we conducted an anonymous online global survey. A total of 176 respondents representing 34 countries participated, including 41% physicians, 49% nurses or midwives, and 6% ultrasound technicians. Most had received OBUS training (72%), reported expertise (60%) and confidence (77%) in OBUS use, and had access to ultrasound (85%). Assessment of gestational age, fetal viability, fetal presentation, and multiple gestation were both the most common OBUS uses and among the most highly prioritized for AI-assisted OBUS development. Most respondents noted ultrasound access was important (84%) and agreed that OBUS improves quality of care (98%) and patient outcomes (97%). Of the 34% expressing reservations associated with using AI-assisted OBUS, healthcare providers not understanding the technology (71%), misdiagnosis (62%), and cost (59%) were the most common. Better understanding the OBUS user, the pregnant individual, and the context, and taking care to ensure responsible, sustainable, and inclusive development and use of AI-assisted OBUS will be critical to successful integration and implementation and to increasing access to OBUS.

**Supplementary Information:**

The online version contains supplementary material available at 10.1038/s41598-025-87284-1.

## Introduction

As part of routine antenatal care, the World Health Organization (WHO) recommends that pregnant individuals have an obstetric ultrasound (OBUS) scan before 24 weeks of pregnancy to estimate gestational age, improve detection of fetal anomalies and multiple pregnancies, reduce induction of labor for post-term pregnancy, and improve a woman’s pregnancy experience^1–3^. For those who did not have an early OBUS scan, the WHO recommends considering a later OBUS scan to identify the number of fetuses, fetal presentation, and placental location. However, because of a variety of logistical, infrastructural, human capacity, and financial challenges, implementing and scaling up this recommendation in many resource-constrained settings has been limited^4,5^. While there are no reliable data on how many pregnant individuals globally receive an OBUS, due to increased portability, durability, usability, and affordability, ultrasound use is increasing, including in low- and middle-income countries (LMIC)^4,6–15^. A versatile and safe imaging technology, ultrasound has a wide variety of applications and uses, with OBUS being the most common indication for ultrasound in LMIC^4,6,13,16,17^.

However, to obtain and interpret OBUS imaging requires trained healthcare providers, and these healthcare providers are not available in all healthcare settings in LMIC. To facilitate healthcare providers to use OBUS to identify and measure various obstetrical and fetal features and conditions, artificial intelligence (AI)-assisted OBUS algorithms to obtain high-quality images and to automatically interpret them are being developed and evaluated^18,19^. Of particular relevance to resource-constrained settings are AI-assisted OBUS algorithms designed to enable novice OBUS users to assess and monitor maternal and fetal health for guiding obstetric care and decision-making. To better understand the current uses of OBUS in LMIC as well as perceptions regarding the potential use of AI-assisted OBUS, we conducted a global online survey.

## Methods

After reviewing the peer-reviewed and published scientific literature to identify common OBUS uses, we developed and piloted a 23-question anonymous online survey in REDCap, separated into five sections, consisting of (1) respondent and (2) healthcare facility demographics, (3) OBUS training and use, (4) OBUS use priorities, and (5) opinions regarding OBUS and AI-assisted OBUS (Appendix 1). Survey respondent and healthcare facility demographics included country, respondent education, current role(s), and information on healthcare facilities and ultrasound equipment. Information on OBUS training, access, and uses was also collected. Respondents were asked to choose the five highest priority OBUS uses in LMIC, taking into account both prevalence/burden and severity. The final section of the survey asked respondents their opinions regarding the importance and value of OBUS in relation to quality of care and patient outcomes, the potential usefulness of AI-assisted OBUS, and any fears or reservations they might have regarding AI-assisted OBUS.

The online survey was open from November 27, 2023 through May 31, 2024. Multiple strategies were employed to target potential survey respondents. We sent the online survey link to colleagues providing care to pregnant individuals in LMIC and those working and providing training in OBUS in LMIC. We contacted potential LMIC respondents via online searches, obstetric conferences, and through in-country professional societies and networks of obstetricians, maternal-fetal medicine specialists, and midwives (specifically, by LMIC region and country). We also contacted corresponding authors of publications identified during our multiple literature reviews, focusing on OBUS use and research in LMIC (specifically, by LMIC country). We chose to include respondents from high-income countries if they had experience with OBUS use in LMIC; when answering questions, respondents were instructed to focus on their experience with and use of OBUS in LMIC. We used the World Bank Atlas method designations for LMIC vs. high-income countries^20^. To improve the response rate, all professional societies and survey respondents contacted were requested to distribute or forward the survey link to their memberships and listservs and to colleagues who provided care to pregnant individuals in LMIC. No sample size calculation was performed.

The cross-sectional survey responses were analyzed descriptively in R version 4.2.2 with simple statistics. Given this was an anonymous survey, no response rate could be calculated. The initial analysis involved examining the data structure and removing entries that lacked data or contained only demographic information. Columns were then analyzed and summary statistics such as range, mean, and standard deviation were calculated where applicable, while counts and percentages were calculated for categorical data. The data were subsequently grouped by respondent education/training and by region, and summary statistics, counts, and percentages were recalculated. The distribution of the most common uses and highest priorities across these groups were then visualized using bar graphs. No statistical tests were utilized. The study and the waiver of informed consent were reviewed by the University of Washington Human Subjects Division, approved, and determined to be exempt due to no greater than minimal risk. All methods were performed in accordance with the relevant guidelines and regulations.

### Role of the funding source

The Bill & Melinda Gates Foundation had no role in the study design, data collection, data analysis, data interpretation, or writing of the manuscript; the co-authors employed by Caption Health/GE Healthcare had input to the study design, data collection, and review of the manuscript.

## Results

**Fig. 1:**
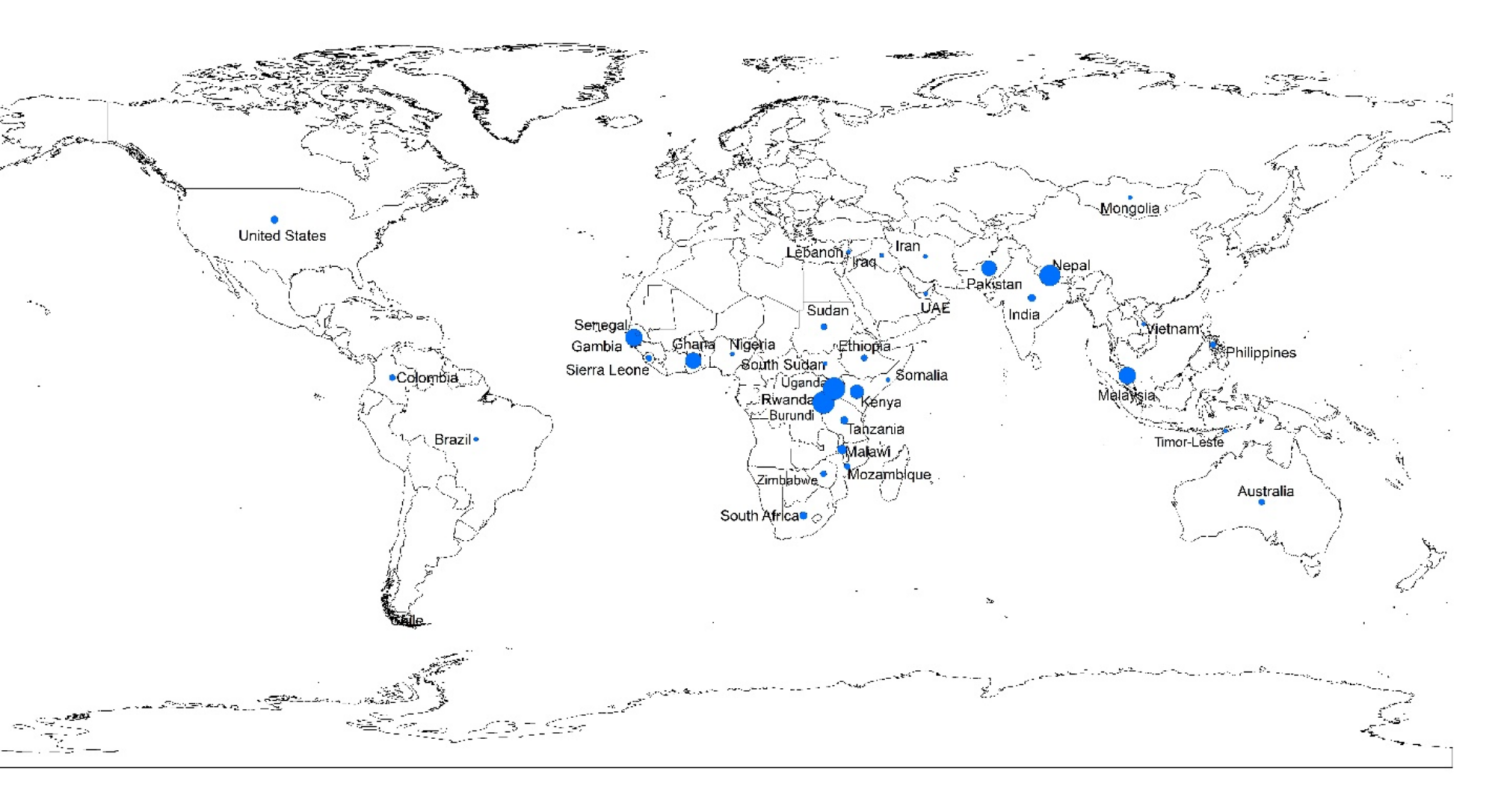
Survey respondents by country. Map created using ArcMap 10.7.1 (ESRI 2019. ArcGIS Desktop: Release 10.7.1. Redlands, CA: Environmental Systems Research Institute).

A total of 176 respondents participated in this online survey, representing 34 countries (Fig. 1), with 96% from LMIC, including 63% from Africa and 32% from Asia (Table 1). The mean age of respondents reporting their age was 41 (range 20 to 74) years and 64% of respondents reporting their sex were female. Of those reporting their highest level of education/training, 41% identified as physicians, 49% as nurses or midwives, and 6% as ultrasound/radiology technicians.

**Table 1:** Survey respondents.

Age (*N* = 169)	
Mean (standard deviation), minimum, maximum	40.8 (11.2), 20, 74
Sex (*N* = 174)	N (%)
Female	112 (64.4)
Male	62 (35.6)
Region (*N* = 168)	N (%)
Africa	106 (63.1)
Asia	54 (32.1)
Other	8 (4.8)
Highest education (*N* = 176)	N (%)
Physician – maternal-fetal medicine specialist	13 (7.4)
Physician – obstetrician	46 (26.1)
Physician – general practitioner	9 (5.1)
Physician – radiologist	1 (0.6)
Physician – other	4 (2.3)
Clinical officer	1 (0.6)
Nurse	35 (19.9)
Midwife	52 (29.5)
Ultrasound/radiology technician	11 (6.2)
Other (e.g., Masters, PhD, Program manager)	4 (2.3)
Current role(s) (*N* = 176)*	N (%)
Physician – maternal-fetal medicine specialist	9 (5.1)
Physician – obstetrician	14 (8.0)
Physician – general practitioner	49 (27.8)
Physician – radiologist	1 (0.6)
Physician – other	3 (1.7)
Clinical officer	3 (1.7)
Nurse	36 (20.5
Midwife	57 (32.4)
Ultrasound/radiology technician	11 (6.2)
Hospital administrator	9 (5.1)
Community health worker	6 (3.4)
Other (e.g., Program advisor, Researcher, Program manager)	3 (1.7)

N = number of respondents who answered the question

*Total percentage for individual question may be over 100% due to “Check all that apply” option for that question on the survey

Among respondents describing their healthcare facility, 61% worked in hospitals, with 69% at a tertiary or academic hospital, and 28% provided care in health centers (Table 2). More respondents worked in healthcare facilities located in urban settings (51%), than in rural (36%) or peri-urban (16%) settings. More respondents worked in publicly funded (77%) than privately funded (31%) healthcare facilities. Access to ultrasound in their current clinical setting(s) was reported by 85% respondents and 11% reported no access to ultrasound. Portable ultrasounds (of which 56% were handheld) were used by 33%, non-portable ultrasounds by 32%, and 35% used both. Probes used included curved (68%), linear (44%), endocavitary (35%), and multi-use (24%). Concerning storage, 45% reported the ability to store OBUS images, with 44% storing on personal devices such as computers, tablets, phones and/or external hard drives, 40% on picture archiving and communication systems, and 22% in electronic health record systems. Of note, only 52% reported their place of work had policies and/or guidelines for OBUS use.

**Table 2:** Healthcare facility and ultrasound equipment.

Healthcare facility type (*N* = 176)*	*N* (%)
Hospital	107 (60.8)
Health center	50 (28.4)
Office or clinic	17 (9.7)
Community health outpost	9 (5.1)
Diagnostic imaging center	4 (2.3)
Other (e.g., academic/training institution, non-governmental organization)	8 (4.5)
Healthcare facility hospital level (*N* = 106)*	N (%)
Specialist/academic/tertiary/referral	73 (68.9)
Provincial	8 (7.5)
District	29 (27.4)
Sub-district	3 (2.8)
Healthcare facility location (*N* = 174)*	N (%)
Urban	88 (50.6)
Peri-urban	28 (16.1)
Rural	63 (36.2)
Other	1 (0.6)
Healthcare facility funding (*N* = 176)*	N (%)
Public	135 (76.7)
Private	55 (31.2)
Other (e.g., non-governmental organization, parastatal, community support)	3 (1.7)
Access to ultrasound (*N* = 175)	N (%)
Yes	149 (85.1)
No	19 (10.9)
Not applicable	7 (4.0)
Number of ultrasound machines (*N* = 129)	
Median, mean (standard deviation), minimum, maximum	2, 3.4 (6.2), 1, 50
Ultrasound machine types (*N* = 148)	N (%)
Portable	49 (33.1)
Not portable	47 (31.8)
Both portable and not portable	52 (35.1)
Handheld portable ultrasound (*N* = 99)	N (%)
Yes	55 (55.6)
No	44 (44.4)
Ultrasound probes used (*N* = 136)*	N (%)
Curved	93 (68.4)
Linear	60 (44.1)
Endocavitary	47 (34.6)
Multi-use probe	33 (24.3)
Phased array	11 (8.1)
Other (e.g., volumetric, unknown)	7 (5.1)
Storage of ultrasound images (*N* = 163)	N (%)
Yes	73 (44.8)
No	70 (42.9)
Not applicable	20 (12.3)
Storage location (*N* = 72)*	N (%)
Personal devices, including computers, tablets, phones, and/or external hard drives	32 (44.4)
Picture archiving and communication systems (PACS)	29 (40.3)
Electronic health record (EHR) systems	16 (22.2)
Hard copy	4 (5.6)
Ultrasound machine	4 (5.6)
Cloud storage	2 (2.8)
Other (e.g., unspecified)	1 (1.4)
Policies/guidelines for obstetric ultrasound use (*N* = 163)	N (%)
Yes	85 (52.1)
No	54 (33.1)
Do not know	24 (14.7)

N = number of respondents who answered the question

*Total percentage for individual question may be over 100% due to “Check all that apply” option for that question on the survey

Of the 72% who received OBUS education and/or training, 56% reported also training others; 28% of respondents reported receiving no OBUS education and/or training (Supplementary Table S1). Upon asking respondents to rate their OBUS expertise, 60% considered themselves very or somewhat experienced, 20% reported being inexperienced or somewhat inexperienced, and 20% reported no experience. When asked about frequency of OBUS use, 58% reported daily use, 15% weekly, 6% monthly or every few months, and 16% rarely or never. Regarding confidence in their ability to use OBUS in clinical care, 77% felt somewhat confident, confident, or very confident in their ability to use OBUS.

**Fig. 2:**
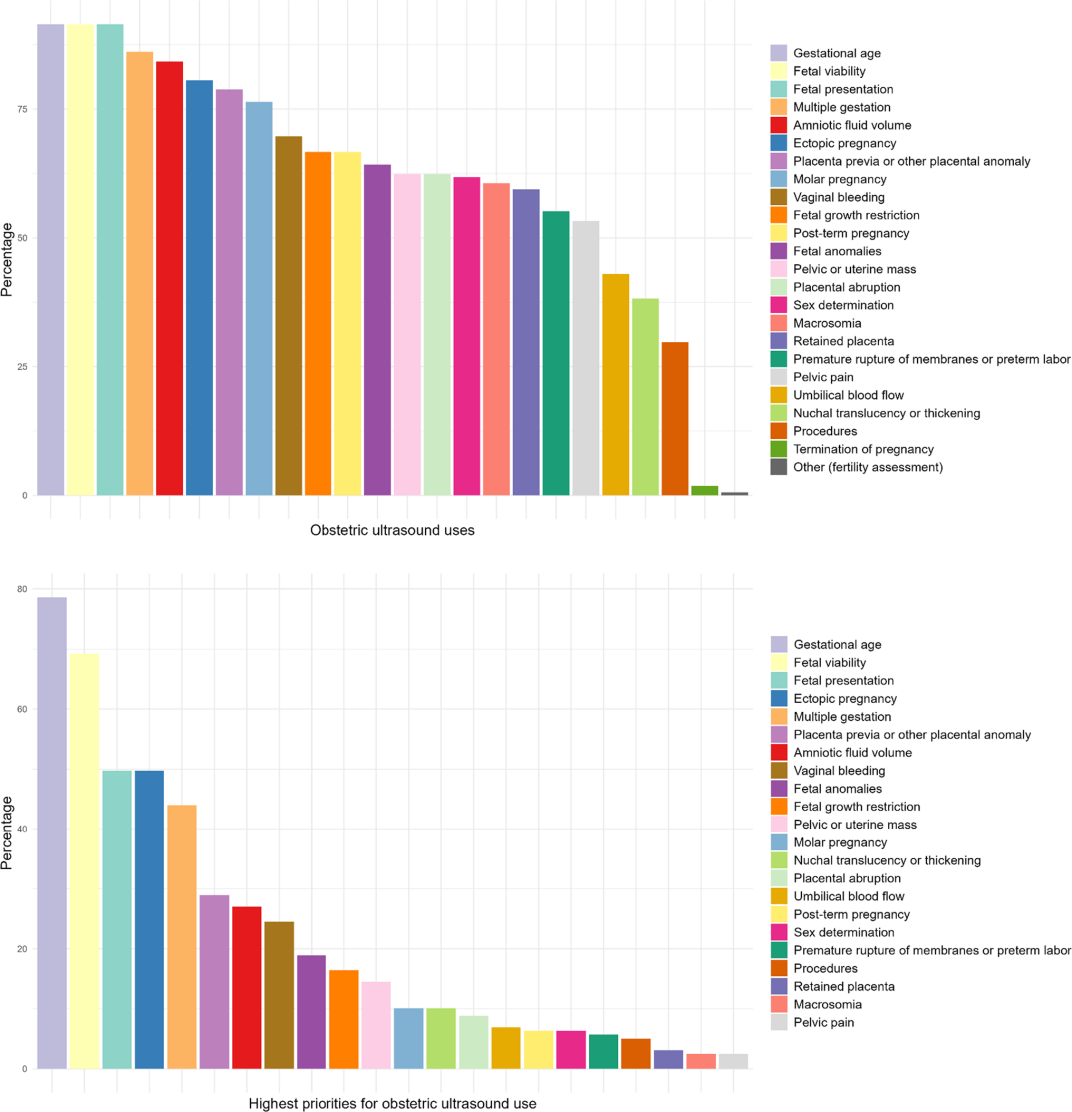
Obstetric ultrasound uses and priorities.

Regarding what they used OBUS for in their clinical setting, when presented with 23 options (including the ability to add other ones), the most commonly selected included assessment of gestational age (92%), fetal viability (92%), fetal presentation (92%), multiple gestation (86%), amniotic fluid volume (84%), ectopic pregnancy (81%), placenta previa or other placental anomaly (79%), and molar pregnancy (76%) (Fig. 2, Supplementary Table S2). When asked to identify only the five highest-priority OBUS uses in their settings, the most commonly chosen were gestational age (79%), fetal viability (69%), ectopic pregnancy (50%), and fetal presentation (50%) (Fig. 2, Supplementary Table S2).

Considering OBUS use by respondent’s highest education/training, over 90% of physicians and ultrasound technicians used OBUS to assess gestational age, fetal viability, ectopic pregnancy, fetal presentation, amniotic fluid volume, and placental previa or other placental anomaly (Table 3). Additionally, maternal-fetal medicine (MFM) specialists and ultrasound technicians utilized OBUS more frequently to assess fetal anomalies and growth restriction (Supplementary Figure S1). MFM specialists (77%) used OBUS for procedures more than general obstetricians (52%), other physicians (36%), and nurses and midwives (13%). Although nurses and midwives made up the largest education/training group of respondents in our survey, compared with physicians, a substantially lower percentage of nurses and midwives utilized OBUS for all use cases except for termination of pregnancy.

**Table 3:** Obstetric ultrasound uses by respondent education/training.

Obstetric ultrasound uses*	Physician, Maternal-fetal medicine specialist (*N* = 13)	Physician, obstetrician (*N* = 46)	Physician, Other (*N* = 14)	Nurse or Midwife (*N* = 77)	Ultrasound/ radiology technician (*N* = 11)
Amniotic fluid volume	13 (100.0)	44 (95.7)	13 (92.9)	56 (72.7)	11 (100.0)
Ectopic pregnancy	12 (92.3)	44 (95.7)	13 (92.9)	50 (64.9)	11 (100.0)
Fetal anomalies	13 (100.0)	35 (76.1)	8 (57.1)	37 (48.1)	11 (100.0)
Fetal growth restriction	13 (100.0)	36 (78.3)	9 (64.3)	39 (50.6)	11 (100.0)
Fetal presentation	13 (100.0)	43 (93.5)	14 (100.0)	66 (85.7)	11 (100.0)
Fetal viability	13 (100.0)	45 (97.8)	13 (92.9)	65 (84.4)	11 (100.0)
Gestational age	13 (100.0)	43 (93.5)	14 (100.0)	66 (85.7)	11 (100.0)
Macrosomia	13 (100.0)	38 (82.6)	9 (64.3)	31 (40.3)	8 (72.7)
Molar pregnancy	11 (84.6)	40 (87.0)	12 (85.7)	52 (67.5)	10 (90.9)
Multiple gestation	13 (100.0)	44 (95.7)	12 (85.7)	59 (76.6)	11 (100.0)
Nuchal translucency or thickening	11 (84.6)	25 (54.3)	5 (35.7)	15 (19.5)	7 (63.6)
Pelvic or uterine mass	10 (76.9)	39 (84.8)	11 (78.6)	30 (39.0)	11 (100.0)
Pelvic pain	11 (84.6)	36 (78.3)	7 (50.0)	22 (28.6)	11 (100.0)
Placenta previa or other placental anomaly	13 (100.0)	44 (95.7)	13 (92.9)	48 (62.3)	10 (90.9)
Placental abruption	9 (69.2)	36 (78.3)	8 (57.1)	39 (50.6)	9 (81.8)
Post-term pregnancy	11 (84.6)	38 (82.6)	11 (78.6)	37 (48.1)	11 (100.0)
Premature rupture of membranes or preterm labor	11 (84.6)	38 (82.6)	6 (42.9)	26 (33.8)	9 (81.8)
Procedures: adjunct to amniocentesis, cervical cerclage placement, external cephalic version,or other procedure	10 (76.9)	24 (52.2)	5 (35.7)	10 (13.0)	0
Retained placenta	10 (76.9)	37 (80.4)	8 (57.1)	32 (41.6)	10 (90.9)
Sex determination	11 (84.6)	33 (71.7)	11 (78.6)	35 (45.5)	10 (90.9)
Termination of pregnancy	0	1 (2.2)	0	2 (2.6)	0
Umbilical blood flow	11 (84.6)	30 (65.2)	9 (64.3)	16 (20.8)	5 (45.5)
Vaginal bleeding	12 (92.3)	41 (89.1)	12 (85.7)	37 (48.1)	11 (100.0)
Other (fertility assessment)	0	1 (2.2)	0	0	0

N = number of respondents who answered the question within each education/training group

*An additional 9 respondents (not included) completed this question and reported obstetric ultrasound was “Not applicable”

Upon considering highest-priority OBUS uses by respondent’s highest education/training, assessment of gestational age was the only use chosen by a majority (> 50%) of respondents across all education/training groups (Table 4). In addition to assessment of gestational age, MFM specialists also identified assessment of multiple gestation (69%), fetal anomalies (62%), ectopic pregnancy (54%), and fetal growth restriction (54%) as the highest OBUS priorities whereas general obstetricians also identified assessment of fetal viability (67%) and ectopic pregnancy (54%), other physicians also chose assessment of fetal viability (69%), and vaginal bleeding (54%), and nurses and midwives also selected assessment of fetal viability (72%), fetal presentation (65%), and multiple gestation (56%) as the highest OBUS priorities (Supplementary Figure S2). Both the most common and the highest-priority OBUS uses were similar when comparing the respondents from Africa vs. Asia vs. Other (Supplementary Table S3).

**Table 4:** Obstetric ultrasound use priorities by respondent education/training.

Highest priority obstetric ultrasound uses	Physician, Maternal-Fetal Medicine Specialist (*N* = 13)	Physician, Obstetrician (*N* = 43)	Physician, Other (*N* = 13)	Nurse or Midwife (*N* = 75)
Amniotic fluid volume	0	11 (25.6)	4 (30.8)	26 (34.7)
Ectopic pregnancy	7 (53.8)	23 (53.5)	4 (30.8)	37 (49.3)
Fetal anomalies	8 (61.5)	11 (25.6)	1 (7.7)	7 (9.3)
Fetal growth restriction	7 (53.8)	13 (30.2)	2 (15.4)	2 (2.7)
Fetal presentation	3 (23.1)	12 (27.9)	6 (46.2)	49 (65.3)
Fetal viability	5 (38.5)	29 (67.4)	9 (69.2)	54 (72.0)
Gestational age	8 (61.5)	31 (72.1)	12 (92.3)	61 (81.3)
Macrosomia	0	2 (4.7)	2 (15.4)	0
Molar pregnancy	2 (15.4)	3 (7.0)	3 (23.1)	8 (10.7)
Multiple gestation	9 (69.2)	12 (27.9)	3 (23.1)	42 (56.0)
Nuchal translucency or thickening	4 (30.8)	4 (9.3)	1 (7.7)	3 (4.0)
Pelvic or uterine mass	1 (7.7)	13 (30.2)	1 (7.7)	6 (8.0)
Pelvic pain	0	1 (2.3)	1 (7.7)	1 (1.3)
Placenta previa or other placental anomaly	4 (30.8)	15 (34.9)	1 (7.7)	22 (29.3)
Placental abruption	0	2 (4.7)	0	11 (14.7)
Post-term pregnancy	0	2 (4.7)	3 (23.1)	4 (5.3)
Premature rupture of membranes or preterm labor	0	2 (4.7)	0	6 (8.0)
Procedures: adjunct to amniocentesis, cervical cerclage placement, external cephalic version, or other procedure	4 (30.8)	2 (4.7)	0	2 (2.7)
Retained placenta	0	1 (2.3)	1 (7.7)	3 (4.0)
Sex determination	0	2 (4.7)	2 (15.4)	6 (8.0)
Umbilical blood flow	1 (7.7)	7 (16.3)	1 (7.7)	2 (2.7)
Vaginal bleeding	2 (15.4)	10 (23.3)	7 (53.8)	13 (17.3)

N = number of respondents who answered the question within each education/training group

The majority of respondents noted that access to ultrasound was very important (80%) or important (4%), with the remainder (16%) believing access was very unimportant (Table 5). Among respondents, the great majority either strongly agreed or agreed that OBUS improves quality of care (98%) and patient outcomes (97%). When asked about the potential usefulness of AI-assisted OBUS in the care of pregnant patients, 53% believed it would be always or frequently useful, 38% sometimes useful, and 9% infrequently or never useful. When queried about any fears or reservations associated with using AI-assisted OBUS in a clinical setting, two-thirds said they had none. Of those expressing fears or reservations, healthcare providers not understanding the technology (71%), misdiagnosis (62%), cost (59%), patients not understanding the technology (47%), malpractice (43%), and misuse (43%) were the most common.

**Table 5:** Perceptions of obstetric ultrasound use and artificial intelligence assistance.

Ultrasound access importance (*N* = 160)	*N* (%)
Very important	128 (80.0)
Important	6 (3.8)
Neither important nor unimportant	0
Unimportant	0
Very unimportant	26 (16.2)
Obstetric ultrasound improves quality of care (*N* = 160)	N (%)
Strongly agree	140 (87.5)
Agree	16 (10.0)
Neither agree nor disagree	1 (0.6)
Disagree	0
Strongly disagree	3 (1.9)
Obstetric ultrasound improves patient outcomes (*N* = 158)	N (%)
Strongly agree	125 (79.1)
Agree	28 (17.7)
Neither agree nor disagree	2 (1.3)
Disagree	0
Strongly disagree	3 (1.9)
Artificial intelligence (AI)-assisted obstetric ultrasound potential usefulness (*N* = 156)	N (%)
Always	43 (27.6)
Frequently	40 (25.6)
Sometimes	59 (37.8)
Infrequently	4 (2.6)
Never	10 (6.4)
Fears or reservations associated with AI-assisted obstetric ultrasound (*N* = 154)	N (%)
No	101 (65.6)
Yes	53 (34.4)
Fears or reservations elaborated (*N* = 53)*	N (%)
Healthcare providers not understanding the technology	38 (71.7)
Misdiagnosis	33 (62.3)
Cost	31 (58.5)
Patients not understanding the technology	25 (47.2)
Malpractice	23 (43.4)
Misuse	23 (43.4)
Leadership/administration not supporting use	11 (20.8)
Confusion about relationship between AI and obstetric ultrasound	2 (3.8)

N = number of respondents who answered the question

*Total percentage for individual question may be over 100% due to “Check all that apply” option for that question on the survey

## Discussion

Informed by 176 respondents from 34 countries and including MFM specialists, general obstetricians, other physicians, nurses, midwives and ultrasound technicians, our global online survey showed that OBUS is used in LMIC for many reasons in the care of pregnant individuals. Most survey respondents reported access within their healthcare settings (85%), and that they had received OBUS education and/or training (72%); although only 52% reported their place of work had policies and/or guidelines for OBUS use. Notably, while almost all respondents believed that OBUS is important for patient care, agreeing or strongly agreeing that OBUS improves both quality of care and patient outcomes, and the majority felt that access to ultrasound is important, 16% asserted access was very unimportant. While surprising, this could potentially indicate that these respondents felt they could still do their jobs without the use of OBUS.

Overall, the most common uses of OBUS in LMIC were for assessment of gestational age (92%), fetal viability (92%), fetal presentation (92%), multiple gestation (86%), amniotic fluid volume (84%) and ectopic pregnancy (81%). Our survey found that OBUS use cases varied by respondent education/training level, although not greatly by region (Africa vs. Asia vs. Other). Perhaps due to less access, different training, and/or their job responsibilities, a substantially lower percentage of nurses and midwives than physicians utilized OBUS for most use cases. Despite OBUS being widely viewed as a critical part of antenatal care, ultrasound and/or healthcare providers trained in OBUS use are not always available or accessible in LMIC settings, and there are many pregnant individuals across LMIC who do not receive an OBUS^5,15^. Access inequity has emerged as a major obstacle to receiving the WHO-recommended OBUS^21–24^.

To help address these constraints and enable lesser-trained healthcare providers to use OBUS, AI-assisted OBUS products are currently being developed, with the goal of increasing equity and ensuring all pregnant individuals have access to OBUS^18,25–29^. AI-assisted OBUS has the potential to improve the accessibility, accuracy, reliability, generalizability, quality, effectiveness, speed, and delivery of antenatal healthcare services and health outcomes. As well as expanding access to services in resource-constrained, rural, or remote LMIC settings without highly trained healthcare providers with specialized imaging expertise, AI-assisted OBUS could have important cost savings and improve equity of care^30^.

When asked to select which AI-assisted OBUS use cases, considering burden and severity, were highest priority for development in LMIC, no single OBUS use case was prioritized by all respondents. Yet, there was some consensus, and the most commonly prioritized were assessment of gestational age (79%), fetal viability (69%), ectopic pregnancy (50%), fetal presentation (50%), and multiple gestation (44%), generally similar to what was reported as the most common uses. Although potential use of AI-assisted OBUS in patient care was viewed favorably by the majority of respondents, 34% of respondents did note some fears or reservations, specifically around not understanding the technology, misdiagnosis, and cost. While exciting and promising innovations such as less expensive, handheld and AI-assisted OBUS technologies are in development, these concerns need to be anticipated and addressed.

Several limitations and challenges need to be overcome for AI-assisted OBUS to become an effective and useful tool in the care of pregnant individuals. In addition to technology challenges, there are existing or emerging risks such as AI system errors, biases, and data privacy and security breaches that could cause patient harm or propagate health inequities^31–33^. Issues such as healthcare personnel capacity, maintenance, overuse and misuse of OBUS, miscommunication between healthcare providers and patients, patient diagnosis and care management, among others, require thoughtful consideration^4,34^. Although LMIC have the potential to benefit most from AI, they also are the most vulnerable to potential harm. AI-assisted OBUS is a tool and does not substitute for the strengthening of health systems.

Despite attempting to distribute our survey widely, there were subgroups (e.g., ultrasound technicians, clinicians in training) we were not able to optimally reach and may not be adequately represented; the small number of respondents completing the survey was a limitation and may be subject to sampling bias. Furthermore, not all respondents completed answers to all survey questions, and some questions had smaller numbers of responses, further limiting generalizability. The survey also was only available online and in English, and so likely did not include respondents who did not have easy access or did not understand English. The survey did not define nor provide information about AI-assisted OBUS and so this may have been a gap in the survey language. Given the survey format and length, we were not able to delve deeper into certain responses for better understanding. Discussion through interviews and focus group discussions with a broad range of relevant stakeholders would be beneficial to capture more comprehensive and in-depth insights regarding OBUS use, priorities, and perspectives and understanding of AI-assisted OBUS.

## Conclusions

Recommended as part of routine antenatal care for pregnant individuals, OBUS use is increasing in LMIC; however, many still remain without access. The most common use cases – assessment of gestational age, fetal viability, fetal presentation – are also the ones most highly prioritized by these respondents for potential AI-assisted OBUS development. Better understanding the OBUS user, the pregnant individual, and the context, and taking care to ensure responsible, sustainable, and inclusive development and use of AI-assisted OBUS will be critical to successful integration and implementation of this potentially transformational tool and to increasing access to OBUS.

.

## Electronic supplementary material

Below is the link to the electronic supplementary material.

Supplementary Material 1

Supplementary Material 2

Supplementary Material 3

## Data Availability

Access to data will be provided to researchers subject to submission of a research proposal and signing a Data Use Agreement. Interested researchers can request access to the data by contacting the corresponding author.
