# Supplementary material for: A survey of obstetric ultrasound uses and priorities for artificial intelligence-assisted obstetric ultrasound in low- and middle-income countries
Source: Sci Rep. 2025 Jan 31;15:3873. doi: 10.1038/s41598-025-87284-1 (PMC11785756; doi:10.1038/s41598-025-87284-1)
Supplement: Supplementary file 1 — Supplementary Material 1 [file 41598_2025_87284_MOESM1_ESM.docx]

**Supplementary Table S1. Obstetric ultrasound training and use**

| **Obstetric ultrasound education and/or training (N = 174)** | **N (%)** |
| --- | --- |
| Yes  No | 126 (72.4)  48 (27.6) |
| **Train others in obstetric ultrasound (N = 174)** | **N (%)** |
| Yes  No | 97 (55.7)  77 (44.3) |
| **Obstetric ultrasound expertise (N = 173)** | **N (%)** |
| Very experienced/Expert  Somewhat experienced  Somewhat inexperienced  Inexperienced/Novice  No experience | 30 (17.3)  74 (42.8)  17 (9.8)  17 (9.8)  35 (20.2) |
| **Frequency of obstetric ultrasound use (N = 176)** | **N (%)** |
| Daily  Weekly  Monthly  Every few months  Rarely  Never  Not applicable | 102 (58.0)  26 (14.8)  8 (4.5)  2 (1.1)  16 (9.1)  12 (6.8)  10 (5.7) |
| **Obstetric ultrasound confidence (N = 172)** | **N (%)** |
| Very confident  Confident  Somewhat confident  Not confident  Not applicable | 40 (23.3)  57 (33.1)  35 (20.3)  31 (18.0)  9 (5.2) |

N = number of respondents who answered the question.

**Supplementary Table S2. Obstetric ultrasound uses and priorities**

| **Obstetric ultrasound uses (N = 165)*** | **N (%)** |
| --- | --- |
| Gestational age  Fetal viability  Fetal presentation  Multiple gestation  Amniotic fluid volume  Ectopic pregnancy  Placenta previa or other placental anomaly  Molar pregnancy  Vaginal bleeding  Fetal growth restriction  Post-term pregnancy  Fetal anomalies  Pelvic or uterine mass  Placental abruption  Sex determination  Macrosomia  Retained placenta  Premature rupture of membranes or preterm labor  Pelvic pain  Umbilical blood flow  Nuchal translucency or thickening  Procedures: adjunct to amniocentesis, cervical cerclage placement, external cephalic version, or other procedure  Termination of pregnancy  Other (fertility assessment) | 151 (91.5)  151 (91.5)  151 (91.5)  142 (86.1)  139 (84.2)  133 (80.6)  130 (78.8)  126 (76.4)  115 (69.7)  110 (66.7)  110 (66.7)  106 (64.2)  103 (62.4)  103 (62.4)  102 (61.8)  100 (60.6)  98 (59.4)  91 (55.2)  88 (53.3)  71 (43.0)  63 (38.2)  49 (29.7)  3 (1.8)  1 (0.6) |
| **Highest priorities for obstetric ultrasound use (N = 159)†** | **N (%)** |
| Gestational age  Fetal viability  Ectopic pregnancy  Fetal presentation  Multiple gestation  Placenta previa or other placental anomaly  Amniotic fluid volume  Vaginal bleeding  Fetal anomalies  Fetal growth restriction  Pelvic or uterine mass  Molar pregnancy  Nuchal translucency or thickening  Placental abruption  Umbilical blood flow  Post-term pregnancy  Sex determination  Premature rupture of membranes or preterm labor  Procedures: adjunct to amniocentesis, cervical cerclage placement, external cephalic version, or other procedure  Retained placenta  Macrosomia  Pelvic pain | 125 (78.6)  110 (69.2)  79 (49.7)  79 (49.7)  70 (44.0)  46 (28.9)  43 (27.0)  39 (24.5)  30 (18.9)  26 (16.4)  23 (14.5)  16 (10.1)  16 (10.1)  14 (8.8)  11 (6.9)  10 (6.3)  10 (6.3)  9 (5.7)  8 (5.0)  5 (3.1)  4 (2.5)  4 (2.5) |

N = number of respondents who answered the question.

*An additional 9 respondents (not included) completed this question and reported obstetric ultrasound was “Not applicable”.

†Respondents were limited to a maximum of five choices.

**Supplementary Table S3. Obstetric ultrasound uses and priorities by respondent region**

| **Obstetric ultrasound uses*** | **Africa**  **(N = 97)** | **Asia**  **(N = 53)** | | **Other**  **(N = 8)** | |
| --- | --- | --- | --- | --- | --- |
| Amniotic fluid volume | 82 (84.5) | 44 (83.0) | | 7 (87.5) | |
| Ectopic pregnancy | 81 (83.5) | 40 (75.5) | | 6 (75.0) | |
| Fetal anomalies | 69 (71.1) | 28 (52.8) | | 3 (37.5) | |
| Fetal growth restriction | 65 (67.0) | 37 (69.8) | | 3 (37.5) | |
| Fetal presentation | 90 (92.8) | 48 (90.6) | | 8 (100.0) | |
| Fetal viability | 91 (93.8) | 47 (88.7) | | 6 (75.0) | |
| Gestational age | 90 (92.8) | 49 (92.5) | | 7 (87.5) | |
| Macrosomia | 62 (63.9) | 31 (58.5) | | 3 (37.5) | |
| Molar pregnancy | 76 (78.4) | 42 (79.2) | | 3 (37.5) | |
| Multiple gestation | 82 (84.5) | 48 (90.6) | | 6 (75.0) | |
| Nuchal translucency or thickening | 36 (37.1) | 21 (39.6) | | 3 (37.5) | |
| Pelvic or uterine mass | 67 (69.1) | 29 (54.7) | | 3 (37.5) | |
| Pelvic pain | 55 (56.7) | 27 (50.9) | | 3 (37.5) | |
| Placenta previa or other placental anomaly | 77 (79.4) | 42 (79.2) | | 5 (62.5) | |
| Placental abruption | 63 (64.9) | 31 (58.5) | | 3 (37.5) | |
| Post-term pregnancy | 71 (73.2) | 30 (56.6) | | 4 (50.0) | |
| Premature rupture of membranes or preterm labor | 54 (55.7) | 29 (54.7) | | 4 (50.0) | |
| Procedures: adjunct to amniocentesis, cervical cerclage placement, external cephalic version, or other procedure | 21 (21.6) | 21 (39.6) | | 4 (50.0) | |
| Retained placenta | 57 (58.8) | 31 (58.5) | | 6 (75.0) | |
| Sex determination | 70 (72.2) | 23 (43.4) | | 5 (62.5) | |
| Termination of pregnancy | 2 (2.1) | 0 | | 1 (12.5) | |
| Umbilical blood flow | 42 (43.3) | 23 (43.4) | | 3 (37.5) | |
| Vaginal bleeding | 71 (73.2) | 33 (62.3) | | 6 (75.0) | |
| Other (fertility assessment) | 1 (1.0) | 0 | | 0 | |
| **Highest priority obstetric ultrasound uses†** | **Africa**  **(N = 97)** | **Asia**  **(N = 47)** | | **Other**  **(N = 7)** | |
| Amniotic fluid volume | 22 (22.7) | 19 (40.4) | | 2 (28.6) | |
| Ectopic pregnancy | 46 (47.4) | 24 (51.1) | | 2 (28.6) | |
| Fetal anomalies | 15 (15.5) | 13 (27.7) | | 2 (28.6) | |
| Fetal growth restriction | 13 (13.4) | 13 (27.7) | | 0 | |
| Fetal presentation | 44 (45.4) | 25 (53.2) | | 5 (71.4) | |
| Fetal viability | 71 (73.2) | 28 (59.6) | | 4 (57.1) | |
| Gestational age | 81 (83.5) | 31 (66.0) | | 5 (71.4) | |
| Macrosomia | 2 (2.1) | 2 (4.3) | | 0 | |
| Molar pregnancy | 7 (7.2) | 9 (19.1) | | 0 | |
| Multiple gestation | 41 (42.3) | 21 (44.7) | | 2 (28.6) | |
| Nuchal translucency or thickening | 7 (7.2) | 8 (17.0) | | 1 (14.3) | |
| Pelvic or uterine mass | 14 (14.4) | 8 (17.0) | | 0 | |
| Pelvic pain | 2 (2.1) | 0 | | 1 (14.3) | |
| Placenta previa or other placental anomaly | 34 (35.1) | 7 (14.9) | | 2 (28.6) | |
| Placental abruption | 13 (13.4) | 1 (2.1) | | 0 | |
| Post-term pregnancy | 10 (10.3) | 0 | | 0 | |
| Premature rupture of membranes or preterm labor | 7 (7.2) | 1 (2.1) | | 1 (14.3) | |
| Procedures: adjunct to amniocentesis, cervical cerclage placement, external cephalic version, or other procedure | 1 (1.0) | 5 (10.6) | | 2 (28.6) | |
| Retained placenta | 3 (3.1) | | 0 | | 2 (28.6) |
| Sex determination | 8 (8.2) | | 2 (4.3) | | 0 |
| Umbilical blood flow | 5 (5.2) | | 5 (10.6) | | 0 |
| Vaginal bleeding | 31 (32.0) | | 5 (10.6) | | 2 (28.6) |

N = number of respondents who answered the question within each region.

* An additional 9 respondents (not included) completed this question and reported obstetric ultrasound was “Not applicable”.

†Respondents were limited to a maximum of five choices.
