# Supplementary material for: A survey of obstetric ultrasound uses and priorities for artificial intelligence-assisted obstetric ultrasound in low- and middle-income countries
Source: Sci Rep. 2025 Jan 31;15:3873. doi: 10.1038/s41598-025-87284-1 (PMC11785756; doi:10.1038/s41598-025-87284-1)
Supplement: Supplementary file 2 — Supplementary Material 2 [file 41598_2025_87284_MOESM2_ESM.docx]

**Supplementary Figure S1. Most common obstetric ultrasound uses by respondent education/training**


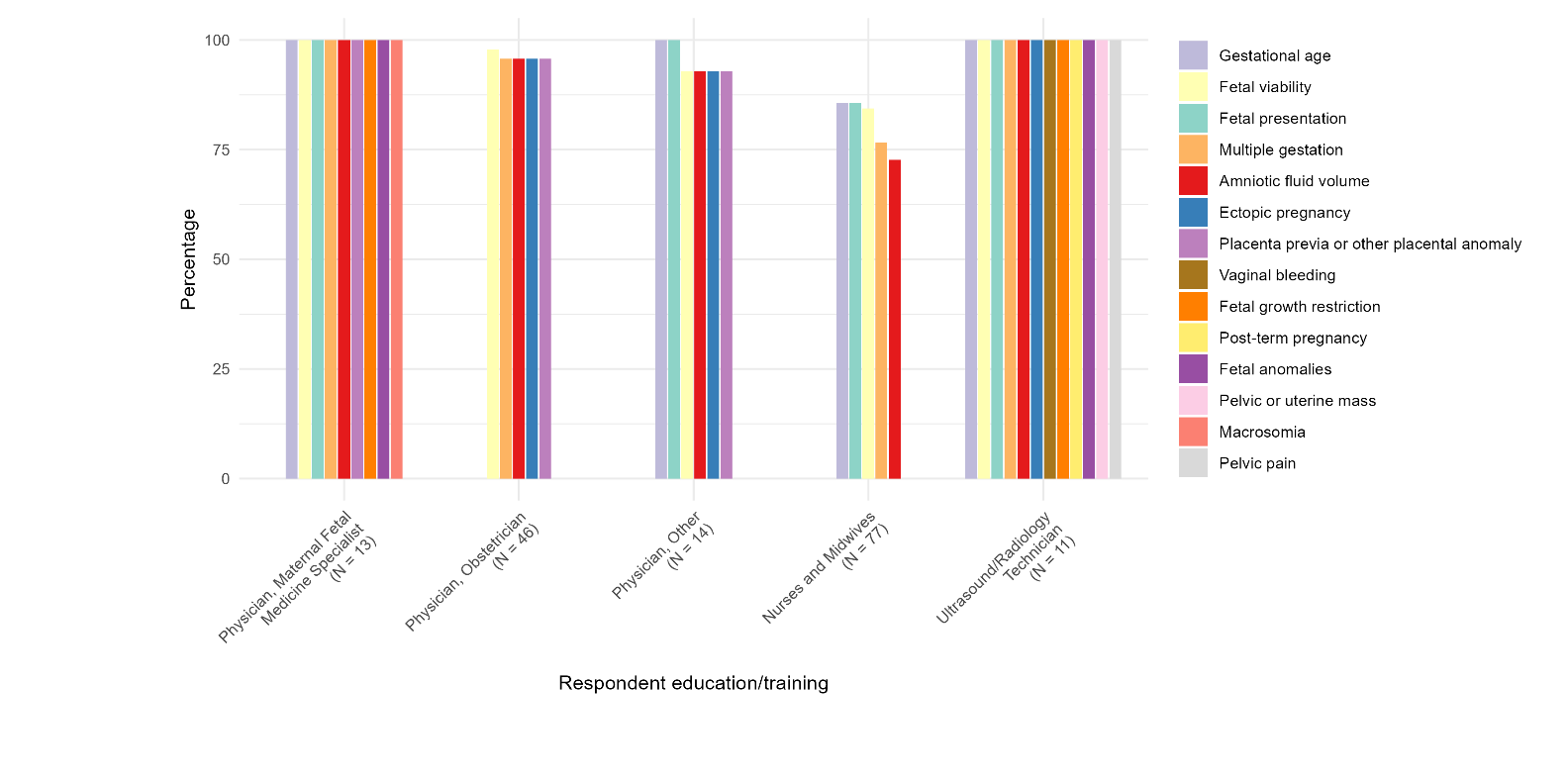


N = number of respondents who answered the question within each education/training group.

**Supplementary Figure S2. Highest obstetric ultrasound use priorities by respondent education/training***
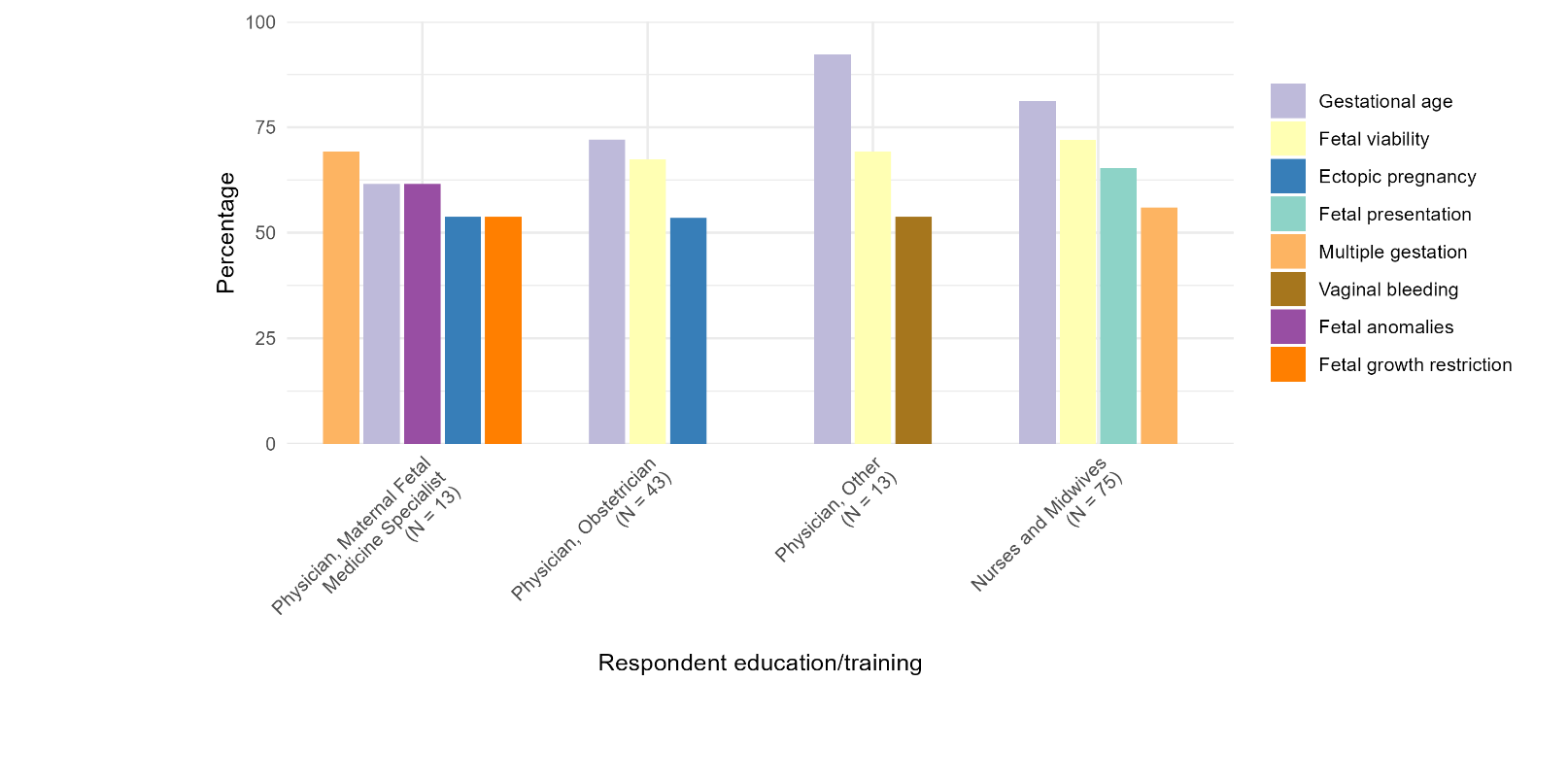


N = number of respondents who answered the question within each education/training group.

*Included only those highest-priority obstetric ultrasound use cases chosen by >50% respondents in each education/training category.
