# Supplementary material for: A survey of obstetric ultrasound uses and priorities for artificial intelligence-assisted obstetric ultrasound in low- and middle-income countries
Source: Sci Rep. 2025 Jan 31;15:3873. doi: 10.1038/s41598-025-87284-1 (PMC11785756; doi:10.1038/s41598-025-87284-1)
Supplement: Supplementary file 3 — Supplementary Material 3 [file 41598_2025_87284_MOESM3_ESM.pdf]

Obstetric Ultrasound

Please complete the survey below.

You are being invited to participate in this online research survey because you take care of pregnant individuals in low- and middle-income countries (LMIC). We are interested in learning more about obstetric ultrasound use in these settings. The goal is to improve the utility and quality of obstetric care in LMIC. To accomplish this, we need input directly from healthcare providers taking care of pregnant individuals in LMIC.

A survey of obstetric ultrasound in low-and middle-income countries

Survey questions will focus on respondent demographics, working location(s), obstetric ultrasound education and training, and obstetric ultrasound use experience, including respondent opinions regarding specific obstetric ultrasound use cases and priorities. The survey will be anonymous and no identifying information will be collected. You can choose to stop the survey at any time; however, once the survey is submitted, the responses cannot be removed. We estimate the survey will take 5-10 minutes to complete. Please remember to SUBMIT your survey at the end.

This research is receiving funding from Caption Health who received funding from Bill & Melinda Gates Foundation for this work. For any questions about the survey, please contact study investigators at ginsburg@uw.edu.

Respondent

1. What is your age? (years) ____________________

2. What is your sex?
   - ◯ Male
   - ◯ Female
   - ◯ Non-binary

3. What country do you live in?

- ◯ Afghanistan
- ◯ Albania
- ◯ Algeria
- ◯ Andorra
- ◯ Angola
- ◯ Antigua and Barbuda
- ◯ Argentina
- ◯ Armenia
- ◯ Australia
- ◯ Austria
- ◯ Azerbaijan
- ◯ The Bahamas
- ◯ Bahrain
- ◯ Bangladesh
- ◯ Barbados
- ◯ Belarus
- ◯ Belgium
- ◯ Belize
- ◯ Benin
- ◯ Bhutan
- ◯ Bolivia
- ◯ Bosnia and Herzegovina
- ◯ Botswana
- ◯ Brazil
- ◯ Brunei
- ◯ Bulgaria
- ◯ Burkina Faso
- ◯ Burundi
- ◯ Cambodia
- ◯ Cameroon
- ◯ Canada
- ◯ Cape Verde
- ◯ Central African Republic
- ◯ Chad
- ◯ Chile
- ◯ China
- ◯ Colombia
- ◯ Comoros
- ◯ Republic of the Congo
- ◯ Democratic Republic of the Congo
- ◯ Costa Rica
- ◯ Cote d'Ivoire
- ◯ Croatia
- ◯ Cuba
- ◯ Cyprus
- ◯ Czech Republic
- ◯ Denmark
- ◯ Djibouti
- ◯ Dominica
- ◯ Dominican Republic
- ◯ East Timor (Timor-Leste)
- ◯ Ecuador
- ◯ Egypt
- ◯ El Salvador
- ◯ Equatorial Guinea
- ◯ Eritrea
- ◯ Estonia
- ◯ Ethiopia
- ◯ Fiji
- ◯ Finland
- ◯ France
- ◯ Gabon
- ◯ The Gambia
- ◯ Georgia
- ◯ Germany
- ◯ Ghana
- ◯ Greece
- ◯ Grenada
- ◯ Guatemala

◯ Guinea
◯ Guinea-Bissau
◯ Guyana
◯ Haiti
◯ Honduras
◯ Hungary
◯ Iceland
◯ India
◯ Indonesia
◯ Iran
◯ Iraq
◯ Ireland
◯ Israel
◯ Italy
◯ Jamaica
◯ Japan
◯ Jordan
◯ Kazakhstan
◯ Kenya
◯ Kiribati
◯ North Korea
◯ South Korea
◯ Kosovo
◯ Kuwait
◯ Kyrgyzstan
◯ Laos
◯ Latvia
◯ Lebanon
◯ Lesotho
◯ Liberia
◯ Libya
◯ Liechtenstein
◯ Lithuania
◯ Luxembourg
◯ Macedonia
◯ Madagascar
◯ Malawi
◯ Malaysia
◯ Maldives
◯ Mali
◯ Malta
◯ Marshall Islands
◯ Mauritania
◯ Mauritius
◯ Mexico
◯ Federated States of
◯ Moldova
◯ Monaco
◯ Mongolia
◯ Montenegro
◯ Morocco
◯ Mozambique
◯ Myanmar (Burma)
◯ Namibia
◯ Nauru
◯ Nepal
◯ Netherlands
◯ New Zealand
◯ Nicaragua
◯ Niger
◯ Nigeria
◯ Norway
◯ Oman
◯ Pakistan
◯ Palau
◯ Panama
◯ Papua New Guinea
◯ Paraguay
◯ Peru
◯ Philippines
◯ Poland

◯ Portugal
◯ Qatar
◯ Romania
◯ Russia
◯ Rwanda
◯ Saint Kitts and Nevis
◯ Saint Lucia
◯ Saint Vincent and the Grenadines
◯ Samoa
◯ San Marino
◯ Sao Tome and Principe
◯ Saudi Arabia
◯ Senegal
◯ Serbia
◯ Seychelles
◯ Sierra Leone
◯ Singapore
◯ Slovakia
◯ Slovenia
◯ Solomon Islands
◯ Somalia
◯ South Africa
◯ South Sudan
◯ Spain
◯ Sri Lanka
◯ Sudan
◯ Suriname
◯ Swaziland
◯ Sweden
◯ Switzerland
◯ Syria
◯ Taiwan
◯ Tajikistan
◯ Tanzania
◯ Thailand
◯ Togo
◯ Tonga
◯ Trinidad and Tobago
◯ Tunisia
◯ Turkey
◯ Turkmenistan
◯ Tuvalu
◯ Uganda
◯ Ukraine
◯ United Arab Emirates
◯ United Kingdom
◯ United States of America
◯ Uruguay
◯ Uzbekistan
◯ Vanuatu
◯ Vatican City (Holy See)
◯ Venezuela
◯ Vietnam
◯ Yemen
◯ Zambia
◯ Zimbabwe

4. What is your highest level of education/training?	◯ Physician - Obstetrician<br>◯ Physician - Maternal-Fetal Medicine Specialist<br>◯ Physician - General Practitioner<br>◯ Physician - Radiologist<br>◯ Physician - Other<br>◯ Clinical officer<br>◯ Nurse<br>◯ Midwife<br>◯ Ultrasound/radiology technician<br>◯ Medical assistant<br>◯ Community health worker<br>◯ Other (Please specify)
Other education (please specify):	__________________________________
5. What is your current role? (Please check all that apply)	☐ Hospital administrator<br>☐ Physician - Obstetrician<br>☐ Physician - Maternal-Fetal Medicine Specialist<br>☐ Physician - General Practitioner<br>☐ Physician - Radiologist<br>☐ Physician - Other<br>☐ Clinical officer<br>☐ Nurse<br>☐ Midwife<br>☐ Ultrasound/radiology technician<br>☐ Medical assistant<br>☐ Community health worker<br>☐ Other (Please specify)
Other role (Please specify):	____________________________
Please feel free to provide any additional comments or explanations to any of the above questions and/or responses.	__________________________________

Healthcare facility (20% Completed)

6. What type of facility do you work in? (Please check all that apply)	☐ Hospital (Please specify level)<br>☐ Health center<br>☐ Office or clinic<br>☐ Diagnostic imaging center<br>☐ Community health outpost<br>☐ Other (Please specify)
Hospital (Please specify and check all that apply):	☐ Specialist/academic/tertiary/referral<br>☐ Provincial<br>☐ District<br>☐ Sub-district<br>☐ Other (Please specify)
Other hospital (Please specify):	__________________________
Other facility not listed above (Please specify):	__________________________
7. Where is your facility located? (Please check all that apply)	☐ Urban<br>☐ Peri-urban<br>☐ Rural<br>☐ Other (Please specify)
Other location (Please specify):	__________________________
8. Do you work in a publicly- or privately-funded facility? (Please check all that apply)	☐ Public<br>☐ Private<br>☐ Other funding source
Other funding source (Please specify):	__________________________
Please feel free to provide any additional comments or explanations to any of the above questions and/or responses.	________________________________

Obstetric ultrasound training and use (40% Completed)

9. Have you had any education and/or training in obstetric ultrasound?	◯ Yes<br>◯ No
10. Have you been involved in training others on the use of obstetric ultrasound?	◯ Yes<br>◯ No
11. How would you rate your obstetric ultrasound expertise?	◯ No experience<br>◯ Inexperienced/Novice<br>◯ Somewhat inexperienced<br>◯ Somewhat experienced<br>◯ Very experienced/Expert
12. How confident are you currently in your ability to use obstetric ultrasound in clinical care?	◯ Not confident<br>◯ Somewhat confident<br>◯ Confident<br>◯ Very confident<br>◯ Not applicable
13. In your current clinical setting, do you have access to ultrasound?	◯ Yes<br>◯ No<br>◯ Not applicable
a. If yes, to how many ultrasound machines?	______________________
b. If yes, what type(s) of ultrasound machines?	◯ Portable<br>◯ Not portable<br>◯ Both portable and not portable
Portable: Is this handheld?	◯ Yes<br>◯ No
c. If yes, what type of ultrasound probes do you have access to ? (Please check all that apply)	☐ Linear<br>☐ Curved<br>☐ Phased Array<br>☐ Endocavitary<br>☐ Multi-use probe<br>☐ Other (Please specify)
Other (Please specify):	______________________
14. How often do you use obstetric ultrasound in your clinical practice?	◯ Daily<br>◯ Weekly<br>◯ Monthly<br>◯ Every few months<br>◯ Rarely<br>◯ Never<br>◯ Not applicable

15. What do you use obstetric ultrasound for in your clinical setting? (Please check all that apply)	☐ Gestational age<br>☐ Ectopic pregnancy<br>☐ Fetal viability<br>☐ Fetal presentation<br>☐ Multiple gestation<br>☐ Vaginal bleeding<br>☐ Pelvic pain<br>☐ Pelvic or uterine mass<br>☐ Molar pregnancy<br>☐ Placenta previa or other placental anomaly<br>☐ Amniotic fluid volume<br>☐ Fetal growth restriction<br>☐ Macrosomia<br>☐ Fetal anomalies<br>☐ Umbilical blood flow<br>☐ Nuchal translucency or thickening<br>☐ Sex determination<br>☐ Post-term pregnancy<br>☐ Premature rupture of membranes or preterm labor<br>☐ Placental abruption<br>☐ Retained placenta<br>☐ Procedures: adjunct to amniocentesis, cervical cerclage placement, external cephalic version, or other procedure<br>☐ Other (Please specify)<br>☐ Not applicable
Other (Please specify):	________________________________
Please feel free to provide any additional comments or explanations to any of the above questions and/or responses.	____

Obstetric ultrasound use priorities (60% Completed) We are considering developing artificial intelligence-assisted obstetrical ultrasound for use by healthcare providers at different levels of the healthcare system in low- and middle-income countries to improve maternal and fetal outcomes. Could you please advise as to the highest priority use cases of obstetric ultrasound in low- and middle-income countries, taking into account both prevalence/ burden and severity?

16. Which are the five highest priority use cases of obstetric ultrasound in your setting? (Please check only 5)

- ☐ Gestational age
- ☐ Ectopic pregnancy
- ☐ Fetal viability
- ☐ Fetal presentation
- ☐ Multiple gestation
- ☐ Vaginal bleeding
- ☐ Pelvic pain
- ☐ Pelvic or uterine mass
- ☐ Molar pregnancy
- ☐ Placenta previa or other placental anomaly
- ☐ Amniotic fluid volume
- ☐ Fetal growth restriction
- ☐ Macrosomia
- ☐ Fetal anomalies
- ☐ Umbilical blood flow
- ☐ Nuchal translucency or thickening
- ☐ Sex determination
- ☐ Post-term pregnancy
- ☐ Premature rupture of membranes or preterm labor
- ☐ Placental abruption
- ☐ Retained placenta
- ☐ Procedures: adjunct to amniocentesis, cervical cerclage placement, external cephalic version, or other procedure
- ☐ Other (Please specify)

Other (Please specify) __________________________________

17. Does your location or place of work have policies and/or guidelines for obstetric ultrasound use?

- ◯ Yes
- ◯ No
- ◯ Don't know

18. Are you able to store ultrasound images?

- ◯ Yes
- ◯ No
- ◯ Not applicable

If yes, where do you store the images? (Please check all that apply)

- ☐ Personal device(s), including computer(s), tablet(s), phone(s), external hard-drive(s)
- ☐ Picture archiving and communication system(s) (PACS)
- ☐ Electronic health record (EHR) system(s)
- ☐ Other (Please specify)

Other (Please specify) __________________________________

Please feel free to provide any additional comments or explanations to any of the above questions and/or responses. __________________________________

Opinions regarding obstetric ultrasound (80% Completed)

19. How important is having access to ultrasound for care of pregnant patients?

- ◯ Very unimportant
- ◯ Unimportant
- ◯ Neither important nor unimportant
- ◯ Important
- ◯ Very important

20. Obstetric ultrasound improves the quality of care for pregnant patients:

- ◯ Strongly disagree
- ◯ Disagree
- ◯ Neither agree nor disagree
- ◯ Agree
- ◯ Strongly agree

21. Obstetric ultrasound improves patient outcomes:

- ◯ Strongly disagree
- ◯ Disagree
- ◯ Neither agree nor disagree
- ◯ Agree
- ◯ Strongly agree

22. Would artificial intelligence-assisted obstetric ultrasound be useful in the care of pregnant patients?

- ◯ Never
- ◯ Infrequently
- ◯ Sometimes
- ◯ Frequently
- ◯ Always

23. Do you have any fears or reservations associated with using artificial intelligence-assisted obstetric ultrasound in a clinical setting?

- ◯ Yes
- ◯ No

If yes, please elaborate. (Please check all that apply)

- ☐ Misdiagnosis
- ☐ Misuse
- ☐ Healthcare providers not understanding the technology
- ☐ Patients not understanding technology
- ☐ Leadership/administration not supporting use
- ☐ Malpractice
- ☐ Cost
- ☐ Other (Please specify)

Other (Please specify):

____
(Other fears/reservations)

Please feel free to provide any additional comments or explanations to any of the above questions and/or responses.

____
